# Case report: Tumor-like mediastinal tuberculous lymphadenitis with paravertebral cold abscess under cumulative immunosuppression: a case highlighting altered host–pathogen interactions

**DOI:** 10.3389/fimmu.2026.1780714

**Published:** 2026-03-06

**Authors:** Xiaoqing Zhou, Zhangjing Sun, Chen Chen, Xin Lv, Ruilin Chen, Zhen Wang

**Affiliations:** 1 The First Affiliated Hospital of Zhejiang Chinese Medical University (Zhejiang Provincial Hospital of Chinese Medicine), Hangzhou, Zhejiang, China; 2 The First School of Clinical Medicine, Zhejiang Chinese Medical University, Hangzhou, Zhejiang, China

**Keywords:** Crohn’s disease, endobronchial ultrasound–guided transbronchial tunneling biopsy, extrapulmonary tuberculosis, host-pathogen interaction, immunosuppression, mediastinal tuberculous lymphadenitis

## Abstract

**Background:**

Tuberculosis (TB) remains a leading opportunistic infection in immunocompromised hosts. Disruption of host–pathogen interactions under cumulative immunosuppression may result in atypical extrapulmonary disease with indolent clinical manifestations and tumor-mimicking radiologic features, leading to substantial diagnostic delay.

**Case presentation:**

A 67-year-old man with Crohn’s disease on cumulative immunosuppressive therapy, including biologics and a Janus kinase inhibitor, developed progressive mediastinal lymphadenopathy and a paravertebral mass with associated vertebral destruction on chest computed tomography, despite prior completion of isoniazid prophylaxis for latent TB infection. The aggressive, tumor-like imaging appearance raised a strong suspicion of metastatic malignancy. Conventional endobronchial ultrasound–guided transbronchial needle aspiration was nondiagnostic. As a salvage diagnostic approach, endobronchial ultrasound–guided tunneling biopsy obtained histological core tissue from a subcarinal lymph node. Although histopathology showed nonspecific fibrous changes without identifiable acid-fast bacilli, Xpert MTB/RIF testing detected *Mycobacterium TB* complex DNA at trace levels. A diagnosis of mediastinal tuberculous lymphadenitis complicated by a paravertebral cold abscess and secondary vertebral osteomyelitis was ultimately established. The patient subsequently showed marked radiological improvement with standard anti-TB therapy.

**Conclusion:**

This case illustrates how cumulative immunosuppression can profoundly alter host immune responses to Mycobacterium TB, resulting in tumor-like extrapulmonary disease and diagnostic ambiguity. Integration of advanced tissue acquisition with molecular testing may be essential for diagnosing TB when disrupted host–pathogen interactions limit conventional diagnostic yield.

## 1 Introduction

Tuberculosis (TB) remains a significant global public health challenge, accounting for a substantial burden of morbidity and mortality worldwide (1). According to the World Health Organization (WHO) 2023 report, the global incidence of TB is estimated at approximately 10.8 million new cases annually (2). Although pulmonary TB is the most common manifestation, extrapulmonary tuberculosis (EPTB) accounts for approximately 15%–25% of reported TB cases worldwide (3). Owing to its nonspecific clinical and radiological features, EPTB is frequently misdiagnosed as malignancy or other inflammatory conditions (4, 5). Accurate and timely diagnosis of TB is therefore essential for appropriate clinical management and improved patient outcomes. Mediastinal tuberculous lymphadenitis (MTL) is uncommon in adults and is more frequently observed in children or individuals with primary TB infection (6, 7). With the widespread and increasing use of tumor necrosis factor (TNF) inhibitors, clinical outcomes in autoimmune diseases have improved substantially; however, the risk of reactivation of latent TB infection has also increased (8). Compared with the general population, TB associated with TNF inhibitor therapy more often presents as disseminated or extrapulmonary disease, including mediastinal lymph node involvement (9). Such misinterpretation may lead to inappropriate treatment strategies, delayed initiation of anti-TB therapy, and an increased risk of TB transmission. Here, we report an older man receiving long-term TNF inhibitor therapy who presented with a mediastinal mass and vertebral destruction. TB was ultimately confirmed by endobronchial ultrasound–guided transbronchial tunneling biopsy (EBUS-TDB) and Xpert MTB/RIF testing. This case underscores the importance of recognizing atypical TB reactivation in immunosuppressed patients. It highlights the value of integrating minimally invasive tissue sampling with molecular diagnostic techniques to facilitate timely diagnosis and treatment.

## 2 Case presentation

**Figure 1:**
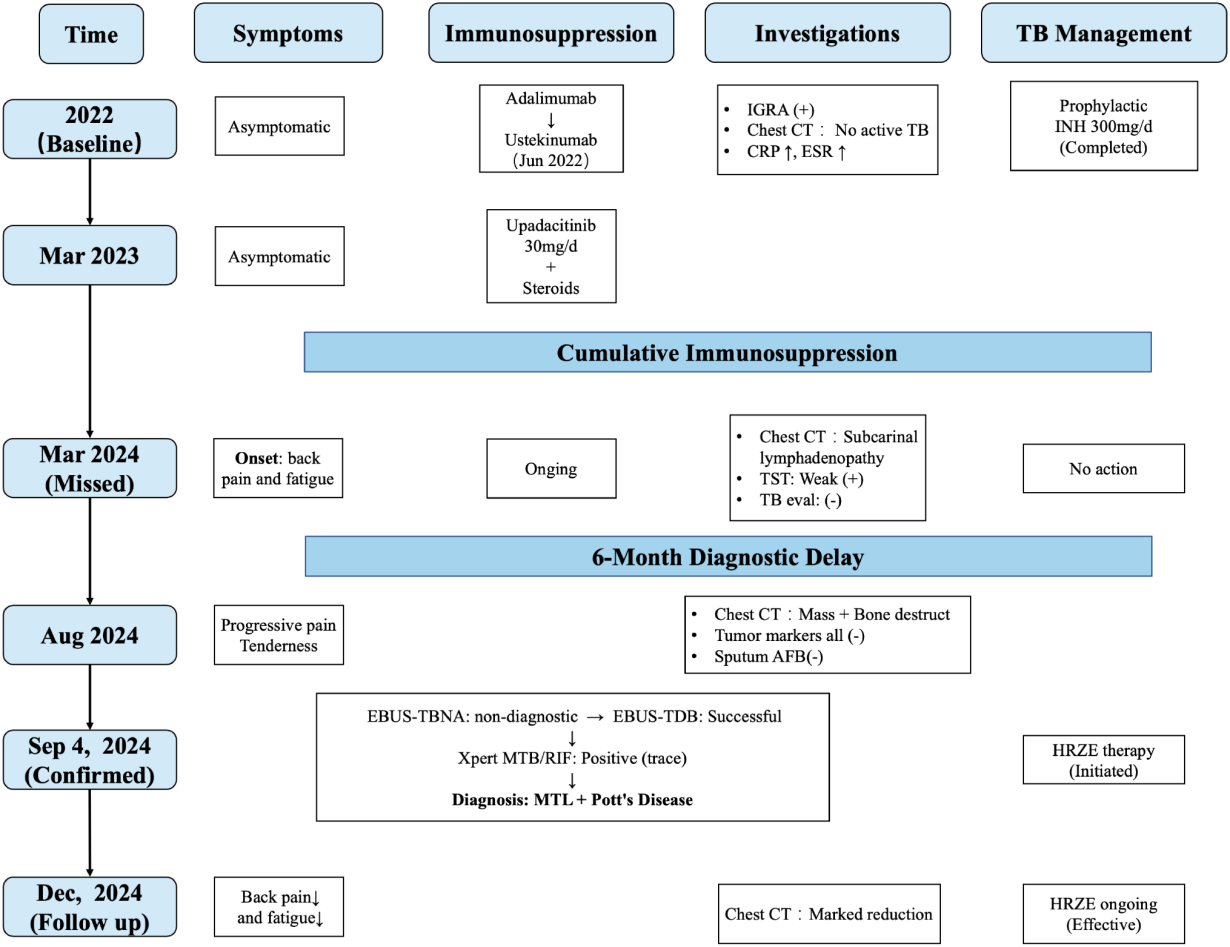
Timeline illustrating the diagnosis and treatment process of the patients. IGRA, interferon-gamma release assay, TB, Tuberculosis; TST, tuberculin skin test, AFB, acid-fast bacilli, EBUS-TBNA, endobronchial ultrasound-guided transbronchial needle aspiration, EBUS-TDB, endobronchial ultrasound–guided transbronchial tunneling biopsy; MTL, Mediastinal Lymph node; HRZE, isoniazid/rifampicin/pyrazinamide/ethambutol.

The clinical timeline of the patient’s disease course is illustrated in **Figure 1**. A 67-year-old man, diagnosed with Crohn’s disease (CD) five years ago, had a surgical history notable for a right hemicolectomy performed 26 years earlier for a colonic “granuloma”. A surveillance colonoscopy in 2022 revealed anastomotic recurrence, classified as a Rutgeerts score of i3. At baseline, the patient was asymptomatic, with no fever, night sweats, weight loss, back pain, or fatigue. Laboratory investigations showed a normal white blood cell count and platelet count, mild anemia (hemoglobin 10^6^ g/L), and elevated inflammatory markers (CRP 37.87 mg/L; ESR 59 mm/h). Liver and renal function tests were otherwise within normal limits. Chest computed tomography revealed scattered small pulmonary nodules and fibrotic changes, with no radiological features suggestive of active tuberculosis. Before initiating biologic therapy, a positive latent TB infection screening result was identified with an interferon-γ release assay (IGRA). The TB antigen–stimulated interferon-γ level was >10.0 IU/mL, and the Nil control was 0.073 IU/mL. As a result, a standard prophylactic course of isoniazid (300 mg/day) was initiated and completed by the patient. Subsequently, the patient experienced a refractory clinical course characterized by secondary loss of response to adalimumab, leading to a therapeutic switch to ustekinumab in June 2022. Owing to persistent disease activity, therapy was further escalated in March 2023 to the Janus kinase (JAK) inhibitor upadacitinib (30 mg/day) in combination with low-dose maintenance corticosteroids. Throughout this period of cumulative immunosuppressive burden, there were no clinical symptoms suggestive of active tuberculosis, including fever, night sweats, weight loss, or respiratory complaints, and no radiological progression or new lesions indicative of active disease on follow-up chest imaging.

**Figure 2:**
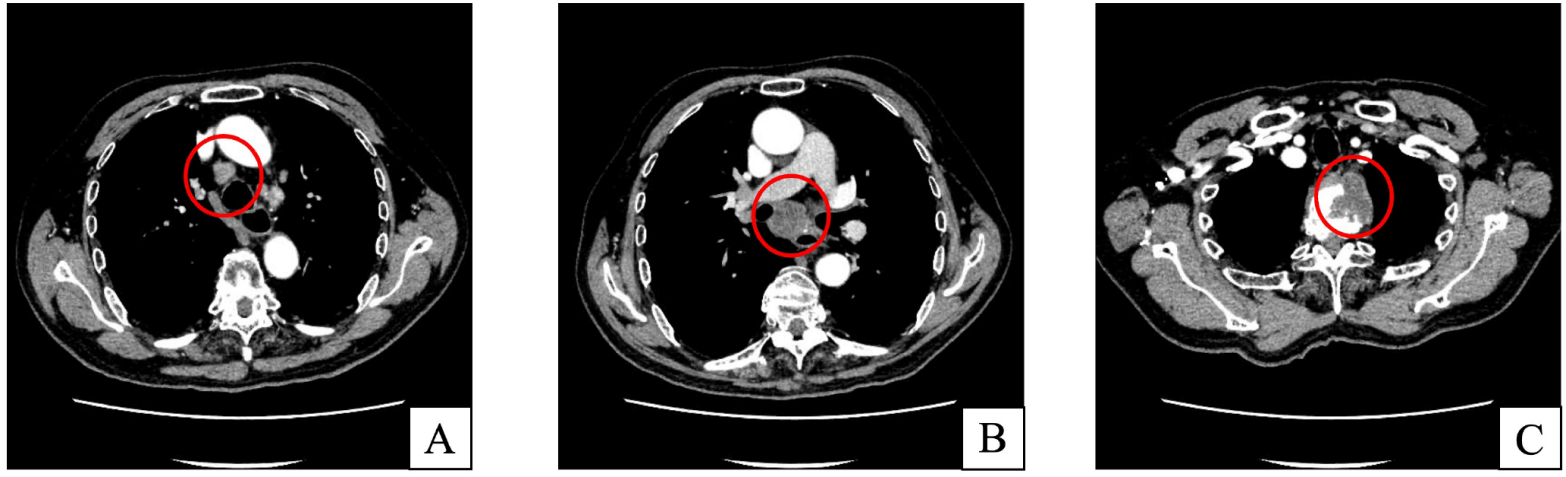
Contrast-enhanced chest CT of the mediastinum. Images obtained on August 29, 2024, show enlarged mediastinal lymph nodes at stations 4R **(A)** and 7 **(B)**. A mass-like paravertebral soft-tissue lesion in the superior mediastinum shows well-circumscribed margins with peripheral rim enhancement and destructive changes involving adjacent vertebral bodies **(C)**.

In March 2024, a surveillance chest computed tomography (CT) scan identified subcarinal lymphadenopathy. Although the tuberculin skin test was weakly positive, specialized TB evaluation found no evidence of active disease. Over the ensuing six months, the patient experienced insidious clinical deterioration, characterized by progressive back pain and fatigue, without constitutional symptoms such as fever, night sweats, or weight loss. During this interval, serial laboratory evaluations were performed. CBC showed no significant leukocytosis or left shift, with overall stable WBC counts. A mild normocytic anemia was present, with Hb fluctuating between 104 and 109 g/L, without progressive deterioration. Inflammatory markers, including CRP and ESR, were persistently elevated with fluctuations, with CRP ranging from 13 to 41 mg/L and ESR from 30 to 70 mm/h, without an acute inflammatory pattern. Routine serum biochemistry results remained within normal limits. No additional imaging studies were obtained during this period. In August 2024, a follow-up contrast-enhanced chest CT (**Figures 2A–C**) revealed progression of subcarinal lymphadenopathy and an upper mediastinal paravertebral mass with adjacent osteolytic vertebral destruction.

These imaging findings raised a strong suspicion of malignancy, particularly metastatic disease. Given the aggressive radiological features, metastatic malignancy was the primary diagnostic consideration; however, infectious etiologies remained in the differential diagnosis owing to the patient’s significant cumulative immunosuppressive history. The physical examination was notable for a thoracic deformity and focal tenderness over the affected vertebral levels. Laboratory investigations revealed a systemic inflammatory response, characterized by elevated C-reactive protein (15.57 mg/L) and erythrocyte sedimentation rate (35 mm/h). In contrast, the white blood cell count was within normal limits (7.3 × 10^9^/L) without neutrophilia or left shift. Mild normocytic anemia was present (hemoglobin 117 g/L, MCV 86.6 fL), with preserved platelet counts. In parallel, an extensive tumor marker panel (CEA, AFP, CA19-9, CA125, CA242, CA50, CA72-4, CYFRA21-1, NSE, SCC antigen, PSA, free PSA, and the free/total PSA ratio) yielded entirely normal results, arguing against an underlying metastatic malignancy. Microbiological evaluation demonstrated negative sputum smears for acid-fast bacilli, and sputum culture revealed only normal respiratory flora. A dedicated fungal workup was subsequently performed, including sputum fungal smear, galactomannan assay, and fungal culture, all of which were negative. Serum anti-tuberculosis antibody testing was positive, whereas autoimmune screening (antinuclear antibodies and antineutrophil cytoplasmic antibodies) was negative.

**Figure 3:**
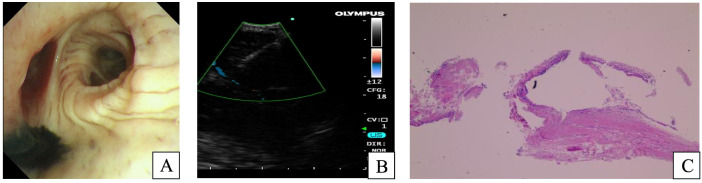
Bronchoscopic and pathological findings. Flexible bronchoscopy on September 4, 2024, revealed focal pigmentation at the orifice of the right middle lobe bronchus **(A)**. Endobronchial EBUS-TBNA was performed on the station 7 lymph node **(B)**. Histopathological examination of the bronchial mucosa showed stromal collagen fiber proliferation with mild chronic inflammatory cell infiltration **(C)**.

Given the lesion’s deep-seated location and the anatomical constraints related to thoracic deformity, a multidisciplinary team deemed percutaneous bone biopsy prohibitively high risk. Consequently, a bronchoscopic approach was selected. Flexible bronchoscopy (**Figures 3A, B**) showed a patent tracheobronchial tree with minimal adherent secretions. Focal mucosal pigmentation was noted at the right middle lobe orifice, where bronchoalveolar lavage was subsequently performed. A convex probe EBUS identified lymphadenopathy at stations 2R, 4R, and 7, with markedly hypoechoic echotexture. Initial EBUS-guided transbronchial needle aspiration (EBUS-TBNA) yielded scant, fragmented material that was nondiagnostic. Subsequently, a salvage EBUS-TDB was performed. A tissue tract was created within the subcarinal (station 7) lymph node via repeated punctures at a single entry point, allowing introduction of biopsy forceps under real-time ultrasound guidance to obtain histological core specimens. The specimens were submitted for histopathological evaluation and microbiological testing, including the Xpert MTB/RIF assay.

**Figure 4:**
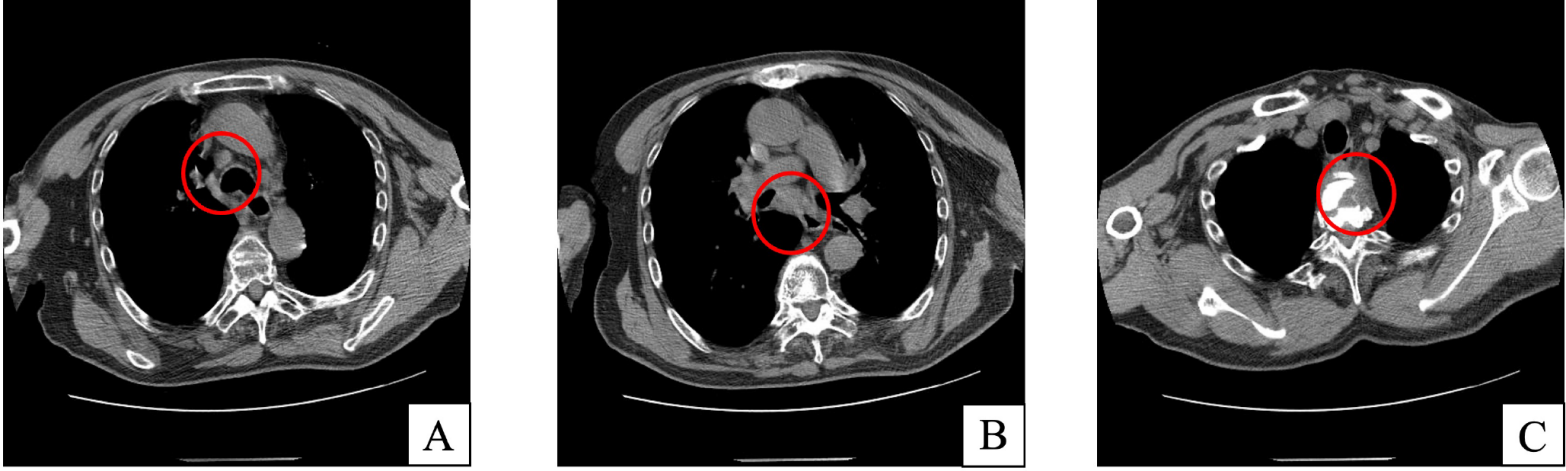
Follow-up chest CT images. Mediastinal window chest CT obtained on December 13, 2024, demonstrated a paravertebral mass-like lesion in the superior mediastinum with adjacent vertebral bone destruction, showing marked improvement compared with the previous examination performed on August 29, 2024 **(A–C)**.

Histopathological examination of the station 7 lymph node biopsy specimen (**Figure 3C**) revealed proliferation of collagenous fibrous tissue in the stromal compartment, accompanied by a small number of chronic inflammatory cells. Immunohistochemical analysis showed low p53 (<5% positive) and Ki-67 (approximately 5% positive) expression, with CK7 (+), TTF-1 (+), Napsin A (−), CK5/6 (+), p63 (+), CD68 (positive in histiocytes), CD34 (highlighting vascular structures), and pan-cytokeratin (+). Special stains showed no acid-fast bacilli, and both periodic acid–Schiff (PAS) and PAS-methenamine silver staining were negative. Liquid-based cytology specimens from lymph node stations 2R, 4R, and 7 were negative for malignancy. Crucially, the Xpert MTB/RIF assay on lymph node tissue detected *Mycobacterium TB* complex DNA at a trace level. Integrating the clinical context, radiological findings, and molecular evidence, a diagnosis of MTL complicated by a paravertebral cold abscess and secondary vertebral osteomyelitis (Pott’s disease) was established. The patient was referred to a specialized TB center and started on standard four-drug anti-TB therapy, including isoniazid, rifampicin, ethambutol, and pyrazinamide. After three months of standard anti-tuberculosis therapy, the patient reported improvement in back pain and fatigue compared with baseline. In parallel, follow-up chest CT performed on December 13, 2024 (**Figures 4A–C**) demonstrated a marked reduction in the size of mediastinal lymph nodes and paravertebral soft-tissue lesions. However, the patient was lost to follow-up after approximately 6 months, and further long-term clinical and radiologic follow-up data could not be obtained.

The patient’s profound distress stemmed from the aggressive radiological mimicry of metastatic malignancy, which he perceived as a terminal outcome. The subsequent molecular confirmation of *Mycobacterium TB* served as a pivotal turning point, redefining the destructive vertebral lesion not as an uncontrollable neoplastic process, but as an opportunistic invasion by a treatable pathogen. Recognizing that his cumulative immunosuppressive burden had created a specific vulnerability to such infection, he viewed the diagnosis as a manageable trade-off in his complex treatment journey. He remained fully compliant with the anti-tuberculosis regimen.

## 3 Discussion

This case illustrates a diagnostically challenging and misleading presentation of extrapulmonary TB in a patient with CD, despite completion of standard latent TB infection prophylaxis; active TB developed during prolonged, sequential immunosuppressive therapy. Predominant mediastinal lymph node involvement with secondary spinal extension produced imaging features that closely mimicked metastatic malignancy, substantially complicating the diagnostic process. These findings emphasize that, in the setting of cumulative immunosuppression, TB should remain a persistent consideration in the differential diagnosis of malignancy-like lesions and highlight the diagnostic value of EBUS-TDB combined with molecular pathogen detection when conventional sampling is inconclusive.

CD is intrinsically associated with dysregulated cell-mediated immunity and impaired mucosal immune surveillance (10), rendering patients susceptible to intracellular pathogens such as *Mycobacterium TB* even before the initiation of immunosuppressive therapy (11). Superimposed on this host background, prolonged and sequential immunomodulatory treatment in inflammatory bowel disease should be regarded as a sustained weakening of anti-mycobacterial immune containment, rather than as isolated risks attributable to individual therapeutic agents (12). Anti–TNF therapy is widely recognized as a key mediator of granuloma formation and maintenance, and its inhibition may compromise the structural containment of latent Mycobacterium tuberculosis. Accordingly, anti-TNF therapy has been consistently associated with a markedly increased risk of tuberculosis reactivation (13, 14). In contrast, IL-12/23 inhibitors, including ustekinumab, generally demonstrate a more favorable tuberculosis safety profile. Although blockade of this pathway impairs Th1 differentiation and interferon-γ production—both critical for macrophage activation—available clinical data suggest a lower incidence of active tuberculosis compared with anti-TNF therapy (14–16). However, sustained suppression of this axis may become clinically relevant in hosts with pre-existing immune dysregulation or prior exposure to multiple immunosuppressive agents. The introduction of Janus kinase inhibitors represents a distinct and mechanistically broader risk. By inhibiting the JAK–STAT signaling pathway, agents such as upadacitinib attenuate cytokine signaling downstream of multiple receptors, including interferon-γ, thereby potentially impairing macrophage-mediated intracellular pathogen control (17). While JAK inhibitors may not disrupt granuloma structure as directly as anti-TNF agents, their widespread suppression of cytokine signaling may facilitate mycobacterial reactivation and dissemination in susceptible hosts, particularly under conditions of cumulative immunosuppression (18, 19).

Importantly, this case illustrates that tuberculosis risk should be interpreted through the lens of cumulative immunosuppressive burden rather than exposure to any single agent in isolation. Sequential therapy involving corticosteroids, anti-TNF agents, IL-12/23 inhibitors, and ultimately a JAK inhibitor combined with maintenance corticosteroids resulted in progressive erosion of host immune containment (20, 21). In this context, although the patient had completed standard isoniazid prophylaxis for latent tuberculosis infection, subsequent profound immunosuppression—especially following initiation of upadacitinib in combination with corticosteroids—likely surpassed a critical immunological threshold, permitting tuberculosis reactivation (22). Furthermore, in immunocompromised hosts, the diagnostic challenge extends beyond initial detection to the assessment of therapeutic efficacy. A delayed therapeutic response is common and can be easily misdiagnosed as treatment failure or drug resistance. To overcome this ambiguity, clinicians should leverage nuanced monitoring advances, such as serial high-resolution imaging, quantitative molecular assays, and emerging artificial intelligence tools (23). These tools provide objective evidence of pathogen clearance even when clinical symptoms lag, thus confirming the correctness of the initial diagnosis and preventing unnecessary changes in management.

The imaging findings in this case substantially increased diagnostic complexity. Progressive mediastinal lymphadenopathy, paravertebral soft-tissue mass formation, and adjacent vertebral destruction are, in routine clinical practice, more commonly suggestive of metastatic malignancy or lymphoproliferative disease, thereby reinforcing a tumor-oriented diagnostic pathway during initial evaluation (24). In contrast, TB involving both the mediastinum and spine is relatively uncommon. It lacks specific imaging characteristics, so it is often not prioritized in the differential diagnosis, particularly in the absence of typical pulmonary involvement. Locally aggressive soft-tissue masses and vertebral destruction, resulting in imaging findings that closely mimic direct tumor invasion or osseous metastasis. This phenotype is closely associated with impaired granuloma formation and compromised local inflammatory control in immunosuppressed states, thereby predisposing TB to manifest as a mass-like lesion rather than a classic infectious process. Consequently, when immunocompromised patients present with mediastinal lymphadenopathy and destructive spinal lesions, reliance on morphological imaging features alone has apparent limitations.

Nevertheless, prior studies have shown that spinal TB (Pott’s disease) and spinal metastases from lung cancer differ in several imaging and clinical features that retain discriminatory value, even in immunocompromised settings, including patterns of lesion distribution, vertebral body and intervertebral disc involvement, characteristics of paravertebral soft-tissue components, and functional imaging findings. Based on previously published literature (23–30) and informed by the imaging features observed in the present case, we systematically compared the major imaging and clinical characteristics of tuberculous vertebral destruction and spinal metastases from lung cancer in **Table 1**. This comparison aims to shift imaging-based differentiation from purely morphological assessment toward a decision-oriented framework, thereby helping clinicians identify imaging features suggestive of an infectious etiology and supporting early consideration of histopathological sampling or microbiological investigation in cases with substantial radiological overlap.

**Table 1:** Clinical and imaging features distinguishing tuberculous spondylitis (Pott’s disease) from spinal metastases associated with lung cancer.

Comparison item	Pott’s disease	Spinal metastases of lung cancer
Patient profile and clinical clues	Occurs in young to middle-aged adults but may also affect the elderly; typically presents with an insidious onset, including fever, weight loss, night sweats, and a history of or concurrent tuberculosis (pulmonary or extrapulmonary). The disease course is usually slow.	More common in middle-aged and elderly patients, often with a known or suspected history of lung cancer. The disease course may be relatively rapid, frequently accompanied by systemic malignancy-related symptoms or radiologic evidence of a primary tumor.
Pattern of spinal involvement	Typically contiguous or skip involvement, often spanning multiple adjacent vertebral levels, consistent with hematogenous dissemination. Anterior vertebral bodies are more commonly involved.	Typically noncontiguous, multifocal involvement consistent with hematogenous dissemination. Posterior elements, including pedicles and laminae, are commonly involved.
Intervertebral disc involvement	Disc involvement is common in early stages, presenting as disc space narrowing or destruction (paradiscal type), although atypical cases with preserved disc spaces may occur. Combined vertebral body–disc involvement is frequent.	Intervertebral discs are usually preserved, as metastases primarily involve vertebral marrow, unless secondary infection or extensive osteolytic destruction of the endplates occurs.
Pattern of vertebral destruction (CT/X-ray)	Fragmentary or patchy destruction across multiple contiguous levels; vertebral collapse, anterior vertebral body erosion, and kyphotic deformity are common.	Predominantly osteolytic lesions with relatively rapid destruction and sharper margins. Involvement of posterior structures is associated with a higher risk of metastatic disease.
Paraspinal/soft-tissue mass	Paravertebral “cold abscesses” and/or epidural extension are common and may be large, typically fluid-containing, thin-walled, and elongated. Calcification within the abscess wall or lesion is not uncommon.	Paraspinal soft-tissue masses are usually solid tumor components; large liquefied abscess-like collections are uncommon.
MRI characteristics	Hypointense on T1-weighted images and hyperintense on T2-weighted images due to marrow edema and inflammation; fluid collections consistent with abscesses may be present. Intervertebral discs often show high signal intensity. Enhancement is typically peripheral or heterogeneous.	Low signal on T1-weighted images with iso- to hyperintense signal on T2-weighted images. Enhancement is usually solid, homogeneous, or nodular. Perilesional edema may be present, but fluid abscesses are rare.
Dynamic contrast-enhanced MRI (DCE-MRI)	Shows a persistent enhancement pattern.	Typically demonstrates rapid initial enhancement followed by washout or a plateau pattern.
FDG PET/CT	Increased FDG uptake; central low uptake may be observed within abscesses. FDG PET/CT carries a risk of false-positive results when differentiating infection from malignancy.	High FDG uptake; useful for identifying the primary tumor and additional metastatic lesions.

Consequently, negative histology cannot reliably exclude tuberculosis in this population. This diagnostic challenge reflects a fundamental immunological principle: the host’s inflammatory phenotype, rather than sampling adequacy, determines histological manifestations of tuberculosis. In the setting of chronic immunosuppression, as seen in this patient receiving sequential anti-TNF, anti-IL-12/23, and JAK inhibition, the capacity to form and maintain structured granulomas—the immunological hallmark of TB containment—is profoundly compromised. Our case illustrates this principle: despite obtaining tissue via EBUS-TDB, initial histology was non-diagnostic, underscoring the limitations of morphology-based approaches when host immunity is severely impaired. Therefore, when host immune surveillance is insufficient to generate diagnostic histological patterns, molecular pathogen detection becomes indispensable. Xpert MTB/RIF applied to bronchoscopic tissue samples provides pathogen-specific confirmation independent of the host’s immunological competence, thereby preventing misdiagnosis in this vulnerable population.

In immunocompromised hosts, impaired cell-mediated immunity fundamentally alters the host response to *Mycobacterium TB*, resulting in poorly formed or absent granulomas with atypical histopathological features. This immunological dysfunction directly impacts diagnostic performance: even advanced tissue sampling techniques such as EBUS-TBNA and EBUS-TDB may yield non-diagnostic results, as the limiting factor is not sample size or architecture preservation, but rather the host’s inability to mount a characteristic inflammatory response (31–34). Consequently, negative histology cannot reliably exclude tuberculosis in this population. This diagnostic challenge reflects a fundamental immunological principle: the host’s inflammatory phenotype, rather than sampling adequacy, determines histological manifestations of tuberculosis. In the setting of chronic immunosuppression, as seen in this patient receiving sequential anti-TNF, anti-IL-12/23, and JAK inhibition, the capacity to form and maintain structured granulomas—the immunological hallmark of TB containment—is profoundly compromised. Our case illustrates this principle: despite obtaining tissue via EBUS-TDB, initial histology was non-diagnostic, underscoring the limitations of morphology-based approaches when host immunity is severely impaired. Therefore, when host immune surveillance is insufficient to generate diagnostic histological patterns, molecular pathogen detection becomes indispensable. Xpert MTB/RIF applied to bronchoscopic tissue samples provides pathogen-specific confirmation independent of the host’s immunological competence, thereby preventing misdiagnosis in this vulnerable population (35, 36).

This case underscores the complex host-pathogen dynamics inherent to cumulative immunosuppression. Notably, it documents the failure of standard prophylaxis, illustrating how *Mycobacterium TB* can breach immune containment when specific signaling pathways are disrupted, ultimately mimicking a neoplastic process. This highlights that TB reactivation is not merely an infection but a reflection of compromised host surveillance. We acknowledge, however, that as an isolated clinical observation, these findings lack the statistical weight of cohort studies. Furthermore, the absence of longitudinal cytokine profiling prevents us from correlating the clinical timeline with specific immunological biomarkers of escape (37, 38).

## 4 Conclusion

The failure of preventive therapy under sequential biologic treatment underscores the persistent fragility of the host-pathogen equilibrium. Since morphological mimicry reflects this altered immune surveillance, clinical reliance must shift from sole imaging to advanced sampling with molecular verification. This integrated approach is essential to facilitate timely management and resolve the complex interplay between host immunosuppression and pathogen persistence.

## Data Availability

The original contributions presented in the study are included in the article/supplementary material. Further inquiries can be directed to the corresponding authors.
